# Mindful practice with medical interpreters

**DOI:** 10.3389/fpsyg.2023.1171993

**Published:** 2023-10-26

**Authors:** Gretchen Roman, Reza Yousefi-Nooraie, Paul Vermilion, Anapaula Cupertino, Steven Barnett, Ronald Epstein

**Affiliations:** ^1^Department of Public Health Sciences, University of Rochester, Rochester, NY, United States; ^2^Department of Family Medicine, University of Rochester, Rochester, NY, United States; ^3^Department of Medicine, Palliative Care Program, University of Rochester, Rochester, NY, United States; ^4^Department of Surgery, University of Rochester, Rochester, NY, United States

**Keywords:** coping, medical interpreters, mindfulness, mindful practice, professional quality of life, resilience, stress, teamwork

## Abstract

**Introduction:**

Medical interpreters experience emotional burdens from the complex demands at work. Because communication access is a social determinant of health, protecting and promoting the health of medical interpreters is critical for ensuring equitable access to care for language-minority patients. The purpose of this study was to pilot a condensed 8-h program based on Mindful Practice^®^ in Medicine addressing the contributors to distress and psychosocial stressors faced by medical sign and spoken language interpreters.

**Methods:**

Using a single-arm embedded QUAN(qual) mixed-methods pilot study design, weekly in-person 1-h sessions for 8 weeks involved formal and informal contemplative practice, didactic delivery of the week's theme (mindfulness, noticing, teamwork, suffering, professionalism, uncertainty, compassion, and resilience), and mindful inquiry exercises (narrative medicine, appreciative interviews, and insight dialog). Quantitative well-being outcomes (mean±SEM) were gathered via survey at pre-, post-, and 1-month post-intervention time points, compared with available norms, and evaluated for differences within subjects. Voluntary feedback about the workshop series was solicited post-intervention via a free text survey item and individual exit interviews. A thematic framework was established by way of qualitative description.

**Results:**

Seventeen medical interpreters (46.2 ± 3.1 years old; 16 women/1 man; 8 White/9 Hispanic or Latino) participated. Overall scores for teamwork (*p* ≤ 0.027), coping (*p* ≤ 0.006), and resilience (*p* ≤ 0.045) increased from pre- to post-intervention and pre- to 1-month post-intervention. Non-judging as a mindfulness component increased from pre- to post-intervention (*p* = 0.014). Compassion satisfaction (*p* = 0.021) and burnout (*p* = 0.030) as components of professional quality of life demonstrated slightly delayed effects, improving from pre- to 1-month post-intervention. Themes such as *workshop schedule, group size, group composition, interactivity, topics to be added or removed*, and *culture* are related to the overarching topic areas of intervention logistics and content. Integration of the findings accentuated the positive *impact of the intervention*.

**Discussion:**

The results of this research demonstrate that mindful practice can serve as an effective resource for medical interpreters when coping with work-related stressors. Future iterations of the mindful practice intervention will further aspire to address linguistic and cultural diversity in the study population for broader representation and subsequent generalization.

## 1. Introduction

Mindfulness and resiliency training in the healthcare workforce has expanded to include many members of the broader care team. Previous literature has investigated the positive effect of mindfulness-based interventions on the health and well-being of medical providers, chaplains, and other allied health professionals (McCracken and Yang, 2008; Irving et al., 2009; Krasner et al., 2009; Geary and Rosenthal, 2011; Beckman et al., 2012; Mehta et al., 2016; Trowbridge and Lawson, 2016; Lomas et al., 2017; Lebares and Hershberger, 2018; Ducar et al., 2020; Grabbe et al., 2021; Epstein et al., 2022); however, few studies have evaluated mindfulness training with medical interpreters (Table 1, note a). Medical interpreters are essential members of the broader care team, serving not only as language interpreters but also as message clarifiers and cultural mediators, all the while navigating the power dynamics between majority and minority cultures (Roat, 1999; Latif et al., 2022). In the absence of an interpreter, language-minority patients were less likely to receive preventive services from their primary care physicians resulting in long-term health implications (McKee et al., 2011). Communication access has been determined a social determinant of health (Smith and Chin, 2012), and thus, providing psychosocial support for the medical interpreter is critical for ensuring equitable access to care (Roman et al., 2022a).

**Table 1:** Glossary of terms used in this study.

**Note**	**Topic**	**Description**
a	Medical interpreter	Medical interpreters bridge communication in medical settings (i.e., inpatient/acute care, outpatient, surgical, urgent care, home care, and skilled nursing/assisted living) between health professionals and staff with patients and families who communicate using different languages. Medical interpreters are fluent in medical terminology across the respective languages. They serve not only as language interpreters but also as message clarifiers and cultural mediators, all the while navigating the power dynamics between majority and minority cultures (Roat, 1999; Latif et al., 2022).
b	Sign language	Sign language is not a universal language (NAD Website, 2023). Sign languages from different countries are their own bona fide visual languages, each with its unique grammatical structure and syntax (Pfau et al., 2012). Two countries with similar spoken languages may have the same or different sign languages. For example, American Sign Language (ASL) is used primarily in the United States and Canada, but British Sign Language is used in Great Britain and Irish Sign Language is used in Ireland.
c	Non-deaf (hearing) sign language interpreters	Hearing sign language interpreters are fluent in the local spoken language and the local sign language.
d	Deaf sign language interpreters	Deaf sign language interpreters offer more nuanced communication to provide access across a wide range of visual languages (NCIEC, 2023). For example, some deaf patients may be from another country, and do not know ASL but are fluent in the sign language of their country of origin. Local resources may not include an interpreter fluent in the deaf patient's sign language. Deaf sign language interpreters work in partnership with the deaf consumer (deaf patient, deaf family of a patient, or deaf caregiver), a hearing medical sign language interpreter, and the health professional(s) to provide a successful communication exchange during the healthcare encounter.
e	Spoken language interpreters	Spoken language interpreters are fluent in the local spoken language and another spoken language.

Medical interpreters and the important roles they play in multilingual, cross-cultural communication often go unnoticed. Medical interpreters have reported feeling isolated and devalued (Latif et al., 2022). They have expressed emotional burdens from the complex demands at work (Butow et al., 2012) and are often confronted with various psychosocial stressors (Park et al., 2017; Lim et al., 2022), which have only become more pressing during the COVID-19 pandemic (Roman et al., 2022a,b). The type of clinic (oncology, psychology, and intensive care), type of medical encounter (new diagnosis of serious illness and end of life), emotional content, the interpreter's role (interpreting bad news), and uncertainty (anticipation and lack of preparation) have been identified as contributors to interpreter distress (Lim et al., 2022). In one study, patient- and system-based stressors, role challenges, and interactions with the medical team were the types of daily stressors experienced by medical interpreters (Park et al., 2017). Patient-based stressors were the largest category and incorporated themes, such as patients who are seriously ill or clinically declining, developing attachments and relationships to patients, and identifying with the patient and family members. System-based stressors included lack of resources, lack of time, and scheduling. Role challenges involved bridging communication between the doctor, patient, and family, translating cultures, making cultural adaptations, maintaining professionalism and accuracy, and breaking bad news. Finally, challenging interactions with the medical team consisted of multiple doctors or caregivers talking simultaneously, having responsibility but no control, not feeling part of the team, and abilities not being respected (Park et al., 2017).

Training and certification vary among medical interpreters due to an array of factors. While some medical interpreters are already certified or working toward certification, not all working medical interpreters are certified unless required by the institution. Professional associations serve as certifying entities and have different minimum education requirements (NBCMI, 2021; CCHI, 2022; RID, 2023). Some medical interpreters must have a minimum of a Bachelor's degree (RID, 2023), whereas others require high school-level education or equivalent and completion of a medical interpreter training for a minimum of 40 h (NBCMI, 2021; CCHI, 2022) or a medical interpreter training course of at least three college or university credit hours (NBCMI, 2021). Even though interpreters undergo training to ensure cultural awareness, language fluency, and competency within specialized settings, the extent to which they receive any professional or on-the-job training to cope with work-related distress is unknown. Some studies (Park et al., 2017) suggest training to cope, however, data on the topic is scant, and medical interpreters' exposure to coping resources and training may vary depending on their working language and the context of their interpreting employment. To promote professionalism in sign language (Table 1, note b) interpreter education, programs seeking to become accredited must maintain mental, physical, and emotional self-care and monitoring standards (Standard 6.1, CCIE, 2019). However, not all interpreting programs are accredited and thus not held accountable for upholding such standards.

In a variety of challenging clinical contexts, medical interpreters have identified the need for training, resources, and support across intrapersonal, interpersonal, and organizational levels. Mindfulness was an intrapersonal resource accessible to some medical interpreters for coping with distress (Lim et al., 2022). A resiliency program was previously piloted with medical interpreters in cancer care (Park et al., 2017). The investigators modified the Relaxation Response Resiliency Program (Park et al., 2013; Mehta et al., 2016) into the Coping and Resiliency Enhancement (CARE) program to meet the unique needs of medical spoken language interpreters and showed pre- to post-participation differences in job satisfaction (Park et al., 2017). This past work demonstrated that the delivery of a relaxation and resiliency program could be successfully modified to a different context and was used to guide the adaptation of a mindful practice program with medical interpreters in the current study.

The purpose of this study was to pilot a condensed 8-h mindful practice program to address the contributors to distress and psychosocial stressors faced by medical sign and spoken language interpreters (Park et al., 2017; Lim et al., 2022). We hypothesized that medical interpreters would experience improved mindfulness, teamwork, stress, coping, resilience, and professional quality of life when responding effectively to the demands at work and that these gains would be sustained at a 1-month follow-up. With the results from this study, study investigators aim to direct greater attention to the occupational well-being of medical interpreters because of their essential roles in bridging communication.

## 2. Materials and methods

This single-arm pilot embedded QUAN(qual) mixed-methods (Schoonenboom and Burke Johnson, 2017) pilot study was approved through the University of Rochester's Research Subject Review Board (STUDY00007212).

### 2.1. Participants

Adults aged 18 years or older were eligible if they predominantly worked as an interpreter in a medical setting and lived within driving distance of the University of Rochester Medical Center's campus in Rochester, NY. Interested participants could be community/freelance, staff, student, and/or video remote interpreters. To reflect the current realities of medical interpreting in the United States, interpreting certification was not required. Any minimum number of medical interpreting hours per week or years of medical interpreting experience was also not required. We included interpreters working within or across medical settings (inpatient/acute care, outpatient, surgical, urgent care, home care, and skilled nursing/assisted living). Previous mindfulness experience was neither encouraged nor prohibited. Participants with previous mindfulness experience or an established mindful practice were asked to adhere to the mindful practices described in the intervention during the time they participated in the study.

We openly recruited non-deaf (hearing) and deaf medical sign language interpreters (Table 1, notes c-d), who were bilingual in English and American Sign Language, and medical spoken language interpreters (Table 1, note e), who were bilingual in English and another spoken language, such as Arabic, Mandarin, or Spanish. We distributed study recruitment materials to local interpreting administrators at medical institutions, interpreter referral agencies, video relay service providers (telecommunication between deaf or hard-of-hearing and hearing participants) who also provide coverage of community medical interpreting requests, teachers at local interpreter education programs with an emphasis on medical interpreting, and not-for-profit associations. We requested these entities to offer recruitment support by dispensing materials to interpreters on their teams, students in their classes, and via their organization's social media and newsletters. Recruitment materials were also posted via the social media accounts of study investigators. Interested medical interpreters clicked a link on the study flier and completed a pre-screening survey. Eligible interpreters received an email (with an information sheet attached for review ahead of time) requesting time to schedule a virtual intake appointment when study procedures and expectations were clearly explained. After completion of the intake appointment, if individuals remained interested in taking part in the study, they were emailed a link to the pre-intervention survey (Cohen et al., 1983; Baer et al., 2006; Smith et al., 2008; Krasner et al., 2009; Muzamil Kumar and Ahmad Shah, 2015; Lomas et al., 2017; Park et al., 2017; ProQOL, 2021; Epstein et al., 2022). Participants who did not meet the inclusion criteria received an email explaining their ineligibility.

The research team worked, respectively, with the education committee and continuing education accreditation and certification maintenance programs at the International Medical Interpreters Association/National Board of Certification for Medical Interpreters (NBCMI 2016), Certification Commission for Healthcare Interpreters (CCHI 2023), and Registry of Interpreters for the Deaf (RID, 2023). Collectively, these certifying entities represented the working languages of Spanish, Cantonese, Mandarin, Russian, Korean, Vietnamese, and sign language. The necessary paperwork was submitted for review, and upon subsequent approval, payment was rendered for support with processing continuing education credits to participants as a means of compensation upon the completion of the intervention. Non-certified interpreters received no equivalent compensation. As this was a pilot study to address the contributors to distress and psychosocial stressors faced by medical interpreters, consistent with current recommendations, we did not conduct a power analysis to determine sample size (Leon et al., 2011). Instead, we used a convenience sample to recruit a number of medical interpreters similar to that reported by Park et al. (2017).

### 2.2. Intervention

Mindful Practice^®^ in Medicine (MPIM) was developed by two physicians at the University of Rochester Medical Center and is an evidence-based educational program designed to build skills and inspire health professionals to thrive, restore joy, and address burnout and distress (Krasner et al., 2009; Epstein et al., 2022; MPIM, 2022). MPIM offers experiential workshops to enhance the self-awareness, emotional intelligence, attentiveness, and compassionate attitudes of health professionals. The goal of MPIM is to advance the quality of interpersonal care in medical settings by improving relationships within healthcare teams and enhancing the resilience and well-being of the healthcare workforce (MPIM, 2022). Past research on MPIM has demonstrated improved and sustained health professional well-being after participation, specifically, reduced burnout and work-related distress, improved empathy, mindfulness, teamwork, job satisfaction, and work engagement, and enhanced personal characteristics for greater delivery of compassionate patient-centered care (Krasner et al., 2009; Epstein et al., 2022).

One of the Co-Directors of MPIM (RE) was an investigator on this study team. Another investigator on the study team (GR) completed MPIM training by participating in the MPIM introductory, core, and facilitator training courses (MPIM, 2022). Cognitive interviews were conducted with the managers and leads of American Sign Language and Spanish patient-care interpreting teams at the University of Rochester Medical Center and insight was shared from collaborators (RH and PV) who have worked with medical interpreters in the Department of Medicine, Palliative Care Program. Interpreting administrators (individuals in positions of administrative leadership) confirmed the potential interest in the topic, shared scheduling preferences and information about interpreter credentials for continuing education credits, offered feedback on proposed incentives, and supported recruitment. Department collaborators helped the research team develop trustworthiness as they had experience disseminating educational information with these teams, and based on such, offered insight into the prominent stressors expressed (i.e., witnessing and supporting patient/family anguish in the face of serious medical information, perceived clinician insensitivity in general or culturally specific clinician insensitivity in communicating that serious medical information, etc.). Based on this input and the relevant literature (Park et al., 2017; Lim et al., 2022), the study team adapted MPIM to address the contributors to distress and stressors faced by medical interpreters. We included challenging interactions with the medical team identified by Park et al. (2017) in the teamwork module (module 3), relevant patient-based stressors in the responding to suffering module (module 4), role challenges in the professionalism module (module 5), and system-based stressors in the uncertainty module (module 6) (Table 2).

**Table 2:** Mindful Practice^®^ in medicine adapted for medical interpreters.

**Week**	**Formal contemplative practice**	**Theme**	**Mindful inquiry exercise**	**Informal contemplative practice**
1	Mindful sitting	Mindfulness	Mindful salon	Grounding, “where are my feet?”
2	Body scan	Noticing	Narrative exercise: meaningful experience	Stop for a moment, take a breath, observe your body, and proceed (STOP)
3	Mindful movement	Teamwork	Narrative exercise: conflict in the work setting	Review and check-in on informal practices from the proceeding weeks
4	Mindful walking	Suffering	Appreciative interview exercise: a moment of suffering	Slow stand and slow sit
5	Mindful sitting	Professionalism	Narrative exercise: maintaining professionalism	“Now, I am aware…”
6	Body scan	Uncertainty	Insight dialog: uncertainty in medical interpreting	Review and check-in on informal practices from the proceeding weeks
7	Mindful movement	Compassion	Appreciative interview exercise: compassion	Loving-kindness, “May you be happy, may you be healthy, may you be safe and free from harm, and may you live life with ease.”
8	Mindful walking	Resilience	Recognize, allow, investigate, and nurture and nourish (RAIN)	Commitment exercise: commit to something positive

The intervention occurred on-site at the University of Rochester Medical Center's campus for 8 weeks with weekly in-person 1-h sessions. Medical sign language interpreters met from July to September 2022 (*n* = 8) and medical spoken language interpreters met from September to November 2022 (*n* = 9). Two study investigators (GR and RE) facilitated the delivery of the mindful practice intervention with sign language interpreters and one study investigator (GR) facilitated the delivery with spoken language interpreters.

**Figure 1:**
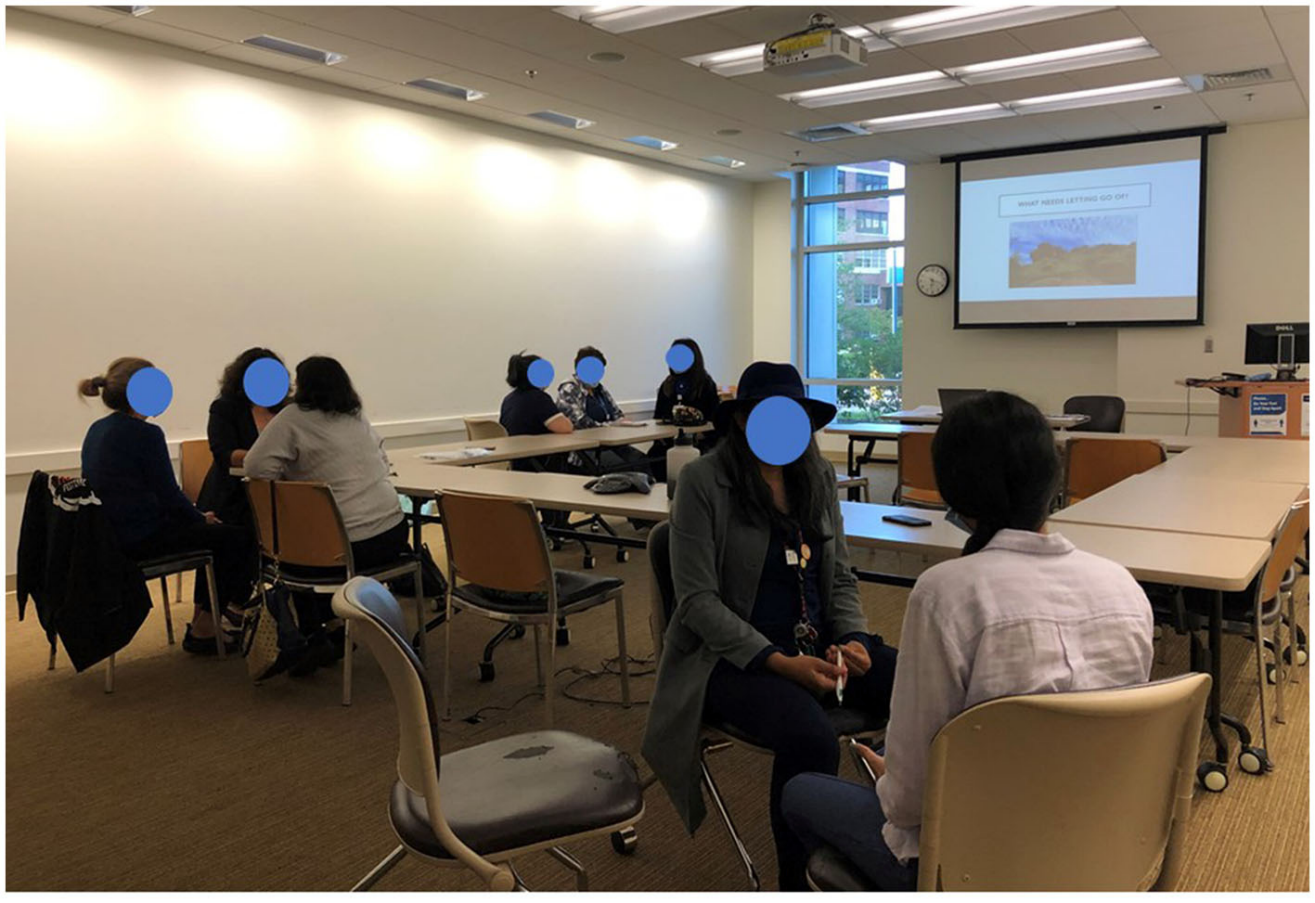
Medical interpreters engaging in a mindful inquiry exercise.

Each session began with a formal contemplative practice designed to enhance the participants' awareness of their thoughts, feelings, and physical sensations and inform them of their moment-to-moment behavior and actions (Epstein and Krasner, 2017). These practices involved mindful sitting, body scan, mindful movement, or mindful walking (Kabat-Zinn, 2013; Epstein and Krasner, 2017; Table 2). After receiving didactic delivery of the week's theme (Table 2), participants engaged in a relevant mindful inquiry exercise working in pairs or small groups (Figure 1). Mindful inquiry exercises involve the contemplation, writing, sharing, and discussion of professional and personal stories (Epstein and Krasner, 2017). These exercises included the mindful salon, narrative medicine, appreciative interviews, insight dialogue, and an exercise known as RAIN, an acronym for recognize, allow, investigate, and nurture and nourish. Based on The World Café (2008), the mindful salon asked reflective questions, such as “What needs to be cultivated?” and “What needs letting go of?” to explore the educational needs of participants. Narrative medicine (Charon, 2001; Epstein and Krasner, 2017; Columbia University Irving Medical Center, 2022) included reflective writing about the participants' clinical experiences or experiences with colleagues, storytelling, deep listening, and reflective questioning (i.e., “what were you unable to see?”, “what are you assuming that might not be true?”, “can you see the same situation with new eyes?”, or “what moved you most about this situation?”). Based on appreciative inquiry (Cooperrider and Whitney, 2005; Epstein and Krasner, 2017), appreciative interviews were used to focus on the participants' positive potential and successes, rather than problems and challenges. Participants also engaged in insight dialog (Kramer, 2007; Epstein and Krasner, 2017; Insight Dialogue Community, 2022), which involved creating personal and interpersonal space in conversation with a colleague (pause/relax/open), being oneself (attune to emergence), and being present (listening deeply/speaking the truth). For RAIN, participants were asked to recognize the thoughts, feelings, and sensations that were happening inside themselves, allow their thoughts, feelings, and sensations to be just as they are, without trying to change them, investigate their inner experience more deeply with curiosity and kindness, and nurture and nourish with self-compassion, offering themselves kindness, support, and understanding (Brach, 2013, 2022; Epstein and Krasner, 2017; Table 2). After each session, participants were instructed in an informal contemplative practice (Epstein and Krasner, 2017; Chozen Bays, 2022) or a brief feasible practice to be implemented when at work and on their own time in between modules. Grounding and loving-kindness were a couple examples of the informal practices shared (Table 2).

### 2.3. Quantitative data collection

Outcomes were collected across three time points (pre-, post-, and 1-month post-intervention) using a collective survey instrument (REDCap; Vanderbilt University, Nashville, TN). The post-intervention data collection occurred immediately upon the completion of the workshop series and the 1-month post-intervention occurred 1-month after completion of the workshop series regardless of whether the participant completed all of the sessions. Our survey included the following measures: Five Facet Mindfulness Questionnaire (FFMQ-15) (Baer et al., 2006; Krasner et al., 2009; Epstein et al., 2022), Organizational Citizenship Behavior Scale (Muzamil Kumar and Ahmad Shah, 2015; Epstein et al., 2022) for teamwork, Perceived Stress Scale (Cohen et al., 1983; Lomas et al., 2017), Part A of the Measure of Current Status (MOCS-A) (Park et al., 2017) for coping, Brief Resilience Scale (BRS) (Smith et al., 2008), and Professional Quality of Life Scale (ProQOL) (Lomas et al., 2017; ProQOL, 2021) (Table 3).

**Table 3:** Description of quantitative well-being outcome measures.

**Outcome variable**	**Tool**	**Description**
Mindfulness	Five Facet Mindfulness Questionnaire (FFMQ-15)	Non-reactivity, acting with awareness, and non-judging sub-scales from the shorter 15-item version of the FFMQ-15 were used to measure mindfulness (Baer et al., 2006; Krasner et al., 2009; Epstein et al., 2022). The non-reactivity sub-scale had three items, which used a 5-point Likert scale (1 = never or very rarely true to 5 = very often or always true). The acting with awareness and non-judging sub-scales also had three items each and used a 5-point Likert scale (1 = very often or always true to 5 = never or very rarely true). Sub-scale scores ranged from 3 to 15 indicating lower to higher non-reactivity, acting with awareness, and non-judging, respectively.
Teamwork	Organizational Citizenship Behavior Scale	The Organizational Citizenship Behavior Scale was used to measure teamwork (Muzamil Kumar and Ahmad Shah, 2015; Epstein et al., 2022). Five types of organizational citizenship behaviors were measured across five sub-scales: altruism, courtesy, sportsmanship, conscientiousness, and civic virtue. With three items per sub-scale, the 15 total items were rated using a 7-point Likert scale (1 = strongly disagree to 7 = strongly agree). Total sub-scale scores ranged from 3 to 21 with higher scores indicating greater altruism, courtesy, sportsmanship, conscientiousness, and civic virtue, respectively. Overall teamwork scores ranged from 15 to 105 with higher scores indicating greater organizational citizenship behavior.
Stress	Perceived Stress Scale	This 10-item instrument (Cohen et al., 1983; Cohen and Williamson, 1988; Lomas et al., 2017) was used to assess an individual's responses to stress and items were rated using a 5-point Likert scale (0 = never to 4 = very often). Total scores ranged from 0 to 40 with 0 to 13 considered as low, 14 to 26 as moderate, and 27 to 40 as high perceived stress.
Coping	Part A – Measure of Current Status (MOCS-A)	This 13-item questionnaire (Park et al., 2017) had relaxation, assertiveness, awareness of tension, and coping confidence sub-scales. Items were rated using a 5-point Likert scale (0 = I cannot do this at all to 4 = I can do this extremely well). The relaxation sub-scale had two items, the awareness to tension and assertiveness sub-scales each had three items, and the coping confidence sub-scale had five items. The average across the relevant items created respective sub-scale scores. Scores ranged from 1 to 4 indicating lower to higher relaxation, assertiveness, awareness of tension, and coping confidence. The sum of the item scores created the overall score, which ranged from 0 to 52 with higher scores reflecting better adaptive coping and stress management.
Resilience	Brief Resilience Scale (BRS)	This 6-item instrument (Smith et al., 2008) used a 5-point Likert scale (positively worded items from 1 = strongly disagree to 5 = strongly agree and negatively worded items from 1 = strongly agree to 5 = strongly disagree) to rate each item. The average across the six items created the overall score. Scores ranged from 1 to 5 indicating lower to higher resilience, respectively.
Professional quality of life	Professional Quality of Life Scale (ProQOL)	This 30-item measure (Lomas et al., 2017; ProQOL, 2021) had compassion satisfaction, burnout, and secondary traumatic stress sub-scales and all items were scored using a 5-point Likert scale (1 = never to 5 = very often). Each sub-scale had 10 items and sub-scale scores ranged from 10 to 50. Scores of 22 or less, 23 to 41, and 42 or higher, respectively, indicated low, moderate, and high compassion satisfaction, burnout, or secondary traumatic stress.

### 2.4. Qualitative data collection

In addition to the quantitative outcome measures, one free text item was included in the post-intervention survey. Participants were asked to voluntarily offer any suggestions about how the intervention could be improved. Also, at the time of the post-intervention survey, participants were offered the opportunity to participate in a voluntary exit interview with a study investigator to elaborate upon any feedback they wanted to share from their time in the workshop series. Each exit interview was performed remotely for 15–50 min and recorded using video conferencing software (Zoom, San Jose, CA). The guide for these individual interviews included questions about intervention logistics, such as “What worked and did not work regarding the workshop series schedule?” and “How did you find the group size?” Questions about intervention content were also included, such as “How did you find the interactivity within each of the sessions (i.e., the formal and informal mindful practices, large group, small group, and paired discussions, as well as the mindful inquiry exercises)?” and “How was the complexity of the content? Do any topics need to be added or removed from the curriculum as it was presented?”

### 2.5. Data analysis

Quantitative data were compiled and qualitative data were manually transcribed. With significance at *p* < 0.05, all quantitative statistical analyses were performed using SPSS (v.29, IBM, Armonk, NY). Descriptive statistics for participant demographics and quantitative outcome variables were calculated (mean ± SEM). Perceived stress and professional quality of life were compared with available normative values (Cohen and Williamson, 1988; ProQOL, 2021). Paired samples *t*-tests and effect size measurements using Cohen's *d* were separately performed to analyze the mindfulness sub-scales, overall teamwork, stress, overall coping, resilience, and professional quality of life sub-scales from pre- to post-intervention and from pre- to 1-month post-intervention for the combined cohort of medical interpreters. While descriptive statistics were reported for the teamwork and coping sub-scales, we elected against analyzing for within-subject differences across time points because of concerns relating to type I errors with multiple comparisons.

A thematic framework matrix was established by way of qualitative description (MaxQDA, Berlin, Germany) for qualitative data analysis (Sandelowski, 2000; Gale et al., 2013; Spencer et al., 2013). Qualitative description used everyday terms and provided a summary of the participant's experience in the workshop series (Sandelowski, 2000). One study investigator (GR) initially coded the data, using a paraphrase or label that described what was interpreted as important in the feedback. Thereafter, two study investigators (GR and RY-N) discussed and debated the initial coding structure and triangulated the identified *topic areas* of improvement or suggestions for the overall study, intervention logistics, and intervention content into *themes* and *sub-themes*. We elected not to create operational definitions after the initial coding, nor did we employ respondent validation because of the straightforward nature of qualitative description. Finally, the same study investigators (GR and RY-N) worked together to integrate the quantitative and qualitative data to emphasize any concordant or discordant findings (Creamer, 2018).

## 3. Results

### 3.1. Participants

Seventeen medical interpreters (46.2 ± 3.1 years old; 16 women/1 man; 8 White/9 Hispanic or Latino) participated. Eight participants were hearing medical sign language interpreters, and nine were medical Spanish interpreters. No deaf medical sign language interpreters participated. Participants had 8.2 ± 2.0 years of medical interpreting experience and worked 26.1 ± 2.9 h/week of medical interpreting. Eleven participants were certified interpreters, and eight reported previous experience with mindfulness. Three withdrew before the start of the intervention citing family reasons unrelated to the study. Interpreters represented two different health systems and worked across congregate living, hospital, outpatient, surgical, urgent care, home care, telehealth, and mobile crisis settings. Four interpreters reported working in two of these settings, six interpreters worked in three settings, four interpreters reported working in four of these settings, one interpreter worked across five settings, one interpreter worked in six settings, and one interpreter reported working across seven settings. Six interpreters worked full-time, two worked part-time, and eight worked per diem or time as reported. One interpreter identified working as a freelance interpreter or independent contractor. Attendance ranged from one to eight sessions (5.3 ± 0.7 sessions). Nine interpreters were able to attend six or more sessions (four interpreters attended all eight sessions, one interpreter attended seven sessions, and four attended six sessions), four interpreters were able to attend between two and five sessions (two attended five sessions, one attended four sessions, and one attended two sessions), and one interpreter only attended one session.

### 3.2. Quantitative outcomes

The survey response rate was 94% (16 out of 17), 82% (14 out of 17), and 82% (14 out of 17) at pre-, post-, and 1-month post-intervention, respectively. Out of 15 possible points for each mindfulness sub-scale, there were no significant within-subject differences (Table 4) across time points for non-reactivity [pre to post: *t*_(13)_ =-1.124, *p* = 0.281, *d* = 0.300; pre to 1-month: t_(13)_ = −1.723, *p* = 0.109, *d* = 0.461] and acting with awareness [pre to post: t_(13)_ = −0.465, *p* = 0.650, *d* = 0.124; pre to 1-month: t_(13)_ = 0.116, p = 0.909, *d* = 0.031]. Within-subject differences significantly improved from pre- to post-intervention for non-judging [t_(13)_ = −2.844, *p* = 0.014, *d* = 0.760]; however, such differences were not maintained from pre- to 1-month post-intervention [t_(13)_ = −2.030, p = 0.063, *d* = 0.543]. Out of 105 possible points for overall teamwork, within-subject differences significantly increased from pre- to post-intervention [t_(13)_ = −2.488, *p* = 0.027, *d* = 0.665] and from pre- to 1-month post-intervention [t_(13)_ = −2.526, *p* = 0.025, *d* = 0.675]. Ranging from 0 to 40 possible points, within-subject scores for stress across time points did not significantly decrease from pre- to post-intervention [t_(13)_ = 1.441, *p* = 0.173, *d* = 0.385]; however, stress demonstrated a moderate effect size (Cohen, 1988; Sawilowsky, 2009) and changed from moderate to low perceived stress (Cohen and Williamson, 1988) when comparing pre- to 1-month follow-up [t_(13)_ = 2.097, *p* = 0.056, *d* = 0.561]. Table 4 conveys the stress values compared with the available norms from the general population (Cohen and Williamson, 1988), as well as the frequencies of participants with high perceived stress and moderate perceived stress or greater. Out of the 52 possible points for overall coping, within-subject increases from pre- to post-intervention [t_(13)_ = −3.290, *p* = 0.006, *d* = 0.879) and from pre- to 1-month post-intervention [t_(13)_ = −3.464, *p* = 0.004, *d* = 0.926] were significant and revealed a strong effect of the mindful practice. The average scores on a 5-point Likert scale across the six items on the BRS significantly improved from pre- to post-intervention [t_(13)_ = −3.202, *p* = 0.007, *d* = 0.856] and from pre- to 1-month post-intervention [t_(13)_ = −2.212, *p* = 0.045, *d* = 0.591]. Ranging from 10 to 50 possible points on each professional quality of life sub-scale, differences within subjects for compassion satisfaction demonstrated a moderate effect size and changed from moderate to high compassion satisfaction (ProQOL, 2021) when comparing pre- to post-intervention [t_(13)_ = −2.085, *p* = 0.057, *d* = 0.557] and significantly increased from pre- to 1-month post-intervention [t_(13)_ = −2.624, *p* = 0.021, *d* = 0.701]. Burnout scores also demonstrated a moderate effect of the mindful practice and changed from low to moderate to low burnout (ProQOL, 2021) when comparing pre- to post-intervention (t_(13)_ = 2.097, p = 0.056, *d* = 0.561) and significantly decreased from pre- to 1-month post-intervention (t_(13)_ = 2.427, p = 0.030, *d* = 0.649). Scores for traumatic stress showed no differences within subjects across time points [pre to post: t_(13)_ = 1.326, *p* = 0.208, *d* = 0.355; pre to 1-month: t_(13)_ = 1.291, *p* = 0.219, *d* = 0.345]. Table 4 conveys the ProQOL values compared with available norms (ProQOL, 2021), the frequency of participants reporting high compassion satisfaction, and the frequencies of participants with moderate burnout or greater and moderate traumatic stress or greater.

**Table 4:** Mean (±SEM) scores and unadjusted differences within subjects across time points for mindfulness, teamwork, stress, coping, resilience, and professional quality of life.

**Outcome**	**Pre-intervention**	**Post-intervention**	**1-month post-intervention**	**pre to post**		**pre to 1-month**	
				***p*-value**	***d***	***p*-value**	***d***
**Mindfulness**							
Non-reactivity	8.88 ± 0.59	9.86 ± 0.61	10.07 ± 0.56	0.281	0.300	0.109	0.461
Acting with awareness	10.94 ± 0.81	11.14 ± 0.67	10.79 ± 0.49	0.650	0.124	0.909	0.031
Non-judging	10.31 ± 0.82	12.43 ± 0.63	12.07 ± 0.60	**0.014** ^*^	0.760	0.063	0.543
**Teamwork**	**77.96** ±**3.40**	**94.36** ±**4.23**	**94.43** ±**4.24**	**0.027** ^*^	**0.665**	**0.025** ^*^	**0.675**
Altruism	16.63 ± 1.32	18.43 ± 0.73	18.57 ± 0.72				
Courtesy	18.44 ± 1.03	19.57 ± 0.40	19.36 ± 0.52				
Sportsmanship	9.38 ± 0.97	9.00 ± 1.20	8.57 ± 1.05				
Conscientiousness	18.06 ± 0.71	18.79 ± 0.37	19.00 ± 0.43				
Civic virtue	14.25 ± 0.86	15.79 ± 0.81	16.00 ± 0.82				
**Stress**	**16.94** ±**2.09**	**13.29** ±**1.26**	**12.21** ±**1.18**	**0.173**	**0.385**	**0.056**	**0.561**
	Moderate perceived stress	Low to moderate perceived stress	Low perceived stress				
Frequency of high perceived stress (≥27 points)	12% (2/17)	0% (0/17)	0% (0/17)				
Frequency moderate perceived stress or greater (≥14 points)	65% (11/17)	35% (6/17)	35% (6/17)				
**Coping**	**26.50** ±**1.55**	**33.50** ±**1.71**	**34.71** ±**2.21**	**0.006** ^*^	**0.879**	**0.004** ^*^	**0.926**
Relaxation	1.66 ± 0.19	2.29 ± 0.18	2.29 ± 0.21				
Assertiveness	1.81 ± 0.19	2.48 ± 0.24	2.74 ± 0.25				
Awareness of tension	2.56 ± 0.24	2.79 ± 0.18	2.83 ± 0.22				
Coping confidence	2.01 ± 0.16	2.63 ± 0.15	2.69 ± 0.16				
**Resilience**	**3.35** ±**0.21**	**4.06** ±**0.19**	**3.80** ±**0.22**	**0.007** ^*^	**0.856**	**0.045** ^*^	**0.591**
**Professional quality of life**							
Compassion Satisfaction	41.69 ± 1.37	43.29 ± 1.28	44.57 ± 1.44	0.057	0.557	**0.021** ^*^	0.701
	Moderate to high compassion satisfaction	High compassion satisfaction					
Frequency of high compassion satisfaction (≥42 points)	53% (9/17)	59% (10/17)	59% (10/17)				
Burnout	21.25 ± 1.68	17.79 ± 1.42	17.50 ± 1.56	0.056	0.561	**0.030** ^*^	0.649
	Low to moderate burnout	Low burnout					
Frequency of moderate burnout or greater (≥23 points)	47% (8/17)	12% (2/17)	12% (2/17)				
Traumatic Stress	20.56 ± 1.39	18.21 ± 1.23	18.07 ± 1.34	0.208	0.355	0.219	0.345
	Low traumatic stress						
Frequency of moderate traumatic stress or greater (≥23 points)	41% (7/17)	12% (2/17)	18% (3/17)				

^*^p < 0.05; *d* = effect size.

### 3.3. Qualitative outcomes

Six out of 17 participants (35%) provided a qualitative response to the free text item on the post-intervention survey, and three out of 17 participants (18%) participated in an individual exit interview. Themes such as *workshop schedule, group size, group composition, interactivity, topics to be added or removed*, and *culture* are related to the overarching topic areas of intervention logistics (Table 5) and intervention content (Table 6). The majority of respondents desired either extending the duration of the session, frequency of the sessions, and/or duration of the overall intervention. Some respondents would not have felt as comfortable sharing in a larger group, whereas others thought a larger group would work well with the understanding that it would require more time and have to cover less material or the material would have to be divided up across more sessions. Respondents valued being with other medical interpreters during the workshop series rather than with interpreters working in non-medical settings and welcomed the differing perspectives of interpreters from other medical organizations and who interpret across different languages. While respondents liked the consistent structure of delivery from session to session, they also liked the different practices with opportunities to work in pairs and take turns in the storyteller and listener roles. Regarding *topics to be added or removed*, one respondent requested *more depth about professionalism* or professional processing of the exposures at work; specifically, more formalized peer debriefing after an assignment. “*We come back from one session and it's just like doing catharsis. I think on a deeper level as professionals, not as human beings complaining (laughing), would be helpful (Medical interpreter #9; Exit interview)*.” The same respondent felt that mindful practice was very inclusive and the intervention allowed them to get in touch with their culture. Another respondent shared that culturally, it felt rushed. “*Hispanics, we like to talk and think out loud. Once we get going, we generally don't say anything unless we're gonna say something, and then we kind of wanna be heard (laughter) (Medical interpreter #12; Exit interview)*.” Regarding *cultural adaptation* of the intervention, more time was suggested to allow participants to process the information and experience. Respondents addressed issues of race, ethnicity, and language of the facilitator. Respondents felt it was important that the facilitator was an interpreter but did not require the facilitator to serve as an interpreter for the same language (a sign language interpreter leading the cohort of sign language interpreters or a Spanish interpreter leading the cohort of Spanish interpreters). Respondents generously shared the personal *impact of the intervention* (Table 6) and humanized the significant quantitative findings (Table 4) for non-judging (mindfulness), teamwork, coping, resilience, compassion satisfaction, and burnout (professional quality of life). “…*this information is freedom. This information is empowerment. And that's what we need to do… every single thing in our lives. We, most of the time, are doubtful or hesitant… and this is just saying, yes you are, and yes you can and this is the way and you are going to have support and that is what this ancient teaching is giving us. I really liked it (Medical interpreter #9; Exit interview)*.” Another respondent stated, “*I did notice, not a change, but an impact. A positive impact of recognizing, of noticing, mostly noticing certain moments… I'm noticing myself, noticing how I'm feeling, noticing the feelings arising, noticing the emotions or the feelings in my body. For me that was huge, I'm like, ‘oh my God, I'm noticing. I'm recognizing, I'm thinking about it.' Before, I wasn't doing that. None of it (Medical interpreter #14; Exit interview)*.”

**Table 5:** Qualitative feedback from study participants about the study overall and intervention logistics.

**Topic area**	**Theme**	**Exemplar quote**	
General feedback about the study overall	Gratefulness and satisfaction	“I am very grateful to have been included in the study. Just the way you and your team wanted to share this practice and this knowledge on how to improve our lives is really valuable… just to say thank you on behalf of the department for this act of inclusion and to put us at this top-of-the-line information and good information about well-being (Medical interpreter #9; Exit interview).”	
	Desired sense of belonging to the organization	“It seems like there's a lot of ‘othering' [a concept relevant to cultural competence] that happens between levels of providers. As interpreters, we feel that. Maybe in some future, when everybody's doing this [mindful practice] and this becomes best practice, combining doctors and interpreters or nurses and interpreters to get those interactive experiences (Medical interpreter #12; Exit interview).”	
Intervention logistics	Attendance	“Virtual option would be helpful (Medical interpreter #3; Free text survey item).”	
		**Sub-theme**	**Exemplar quote**
		Internal motivation	“What allowed me to come every week and not miss a session was the love for myself, the love for my career, and the impact I have in the community (Medical interpreter #9; Exit interview).”
			“Being able to participate in 100% of all the different meetings is probably a particularity of my personality. Early on, I learned if you're gonna sign-up for something, be there (Medical interpreter #12; Exit interview).”
	Workshop schedule	**Exemplar quote**	
		“The schedule and the hours were fine for me. An hour was enough for us to stay focused and do the exercises. I think we were able to manage the time very well. We would like to stay a bit longer just to go deeper into some aspects, but I think 1 hour is fine and after the regular working hours it was fine, once a week every single week (Medical interpreter #9: Exit interview).”	
		“I felt that we ran out of time very often, so I would suggest extending the duration of each session to 90 minutes (Medical interpreter #10; Free text survey item).”	
		“The hour was a perfect thing. You are after work hours, so you are tired. But, since it's only an hour, it's a perfect time, not too long (Medical interpreter #14; Exit interview).”	
		**Sub-theme**	**Exemplar quote**
		Intervention able to build involvement	“I was worried about the 8 weeks for an hour, but after a few weeks, I enjoyed going and even looked forward to it (Medical interpreter #5; Free text survey item).”
		Concerns about the length and burden of the intervention	“Sometimes it was hard to take all the information in and think of a story to share in the right way and in the time allowed. Getting people to commit to 1.5 h a week is harder, but could be a benefit (Medical interpreter #5; Free text survey item).”
		Longer duration or greater frequency	“In terms of frequency, for me personally, it would have been better maybe twice a week.” “If I'm starting to notice now and to be aware, imagine how much more I could do if the research would have lasted 16 weeks or if it wasn't just eight times throughout 8 weeks, but maybe twice a week throughout 8 weeks. I feel like I would have benefitted more and you would have gotten more data and feedback from us. To me, it just felt short (Medical interpreter #14; Exit interview).”
		Suggestions for less topics, more depth	“I think no more than 8 [participants]. If it's more than 8, then it'd have to be longer and divided up even more, because it's so personal and intense in some ways. It would have to be much longer and cover less material (Medical interpreter #12; Exit interview).”
	Group size		
		**Exemplar quote**	
		“The size was very manageable. We had the opportunity to talk in pairs with everybody. Although the topics were different, but we were able to exchange our views and experiences with that size… maybe 14, or 16 is fine (Medical interpreter #9; Exit interview).”	
		“Our group size was perfect, but a larger group could also work well (Medical interpreter #10; Free text survey item).”	
		“I think it was a good group size. I think if it would have been bigger, I would have not felt as comfortable sharing, probably (Medical interpreter #14; Exit interview).”	
	Group composition	“I enjoyed the practices in pairs because we could openly share personal and sometimes very sensitive experiences (Medical interpreter #10; Free text survey item).”	
		“I liked that we always had a different person we were working with. I like that we got to do turn-taking, so that was nice (Medical interpreter #12; Exit interview).	
		**Sub-theme**	**Exemplar quote**
		Organizational diversity	“I would have liked to have the presence of other interpreters. We had an interpreter that was not from the same organization and it was very enriching. She gave very good insights as an individual and as an interpreter. I think to have been in session with sign language interpreters, with interpreters of other languages, would have been a great add on to the activity (Medical interpreter #9; Exit interview).”
		Interpreters from the same setting, but with linguistic diversity	“Definitely other languages make you realize different perspectives…” “There was for sure a benefit to having the same type of medical interpreters in the group. I felt like they were able to familiarize with what you are going through, so there was definitely a benefit. But, personally, since I'm someone who is so curious, I am always fascinated by the different reactions over the same situation (Medical interpreter #14; Exit interview).”
			“So, I think one of the things that made this resonate with me was the comments other interpreters were making. The reason that they resonated with me is because they were working in the same hospital under the same conditions. We have those shared experiences that we can bounce off of each other. As researchers, you would probably get a richer thing if you're seeing, like interpreters that work with schools or interpreters that work for compensation cases… but my concern, I know for myself, would be like, you're saying stuff that I really can't even relate to. I think even more important than the same language is the kind of interpreting that we are doing (Medical interpreter #12; Exit interview).”
	General feedback about logistics	**Exemplar quote**	
		“I'm wondering if the setting, like maybe the circle [of tables and chairs] could be smaller somehow, so we can feel more connected? (Medical interpreter #12; Exit interview).”	

**Table 6:** Qualitative feedback from study participants about intervention content.

**Topic area**	**Theme**	**Exemplar quote**	
Intervention content	Interactivity	“I liked that you had different practices, like ‘STOP [Stop, Take a breath, Observe, Proceed],' ‘May you be happy, healthy, safe and free from harm…', and laughing while tossing the Beanie Babies was very freeing, almost childlike. These are the ones that stuck with me and I was able to use (Medical Interpreter #5; Free text survey item).”	
		“The content each week was different with the same objective… just how to manage ourselves to feel better with ourselves and this way, we will have a better orientation to do what we do, not only in life but as professionals (Medical interpreter #9; Exit interview).”	
	Complexity	“The activities were very well designed for us to understand what we had to do (Medical interpreter #9; Exit interview).”	
		“It was a wonderful experience. The topics were on point, clear, simple, and practical (Medical interpreter #10; Free text survey item).”	
		“I did not feel that the content was complex at all. I felt that it was very easy to understand, very easy to digest, very easy to talk about, very fascinating to me personally. I loved every topic (Medical interpreter #14; Exit interview).”	
	Topics to be added or removed	“I don't feel that any should be erased and I'm not in a position where I know more to say, ‘oh, this one should be added.' (Medical interpreter #14; Exit interview).”	
		**Sub-theme**	**Exemplar quote**
		More depth about professionalism	“No topics needed to be added or removed, but the topic that included professionalism… I think we could have gone deeper and further with more time on that topic. We do a lot of informal curriculum. We talk a lot. All the time. We come back from one session and it's just like doing catharsis. I think on a deeper level as professionals, not as human beings complaining (laughing), would be helpful (Medical interpreter #9; Exit interview).”
	Learning aids	Presentation handouts	“Just the objectives were given and the PowerPoint was from the previous session, which was good… still something to be discovered in the class. That was perfect.” “The PowerPoint should have the letters a bit bigger. In that case, we can even print them out, post it as little posters, and then have it as a reminder (Medical interpreter #9; Exit interview).”
			“For me, the way I learn, I wonder if it would have helped me, to have a copy of the slides in front of me? I have to print everything and look at it, touch it, write over it, and underline, and that helps me learn better and it also somehow helps me make the connection better (Medical interpreter #12; Exit interview).”
		**Exemplar quote**	
		“Not only did you have visual because certain people learn more audio-wise, than visual-wise, but you also had a part where you had a video demonstrating the differences in the [appreciative] interview. You had a mix, so everyone would be able to learn the way they learn (Medical interpreter #14; Exit interview).”	
		“The [appreciative interview] demonstration was really helpful. The slides were good. There was something soothing about most of the slides, which I found enjoyable. But there were times when I felt like there's a lot on that slide, not because it was visually dense, but because there were a lot of important concepts there that I was still trying to digest (Medical interpreter #12; Exit interview).”	
	Culture	“This is a way to get in touch with our culture. Each of us is a piece of culture, our own countries, and I think this is a very inclusive attitude, which is really good in these times when everybody is trying to be in their own self and not share (Medical interpreter #9; Exit interview).”	
		“Culturally, it felt rushed. Hispanics, we like to talk and think out loud. Once we get going, we generally don't say anything unless we're gonna say something, and then we kind of wanna be heard (laughter) (Medical interpreter #12; Exit interview).”	
		**Sub-theme**	**Exemplar quote**
		Cultural adaptation	“I think ensuring spaciousness is enough to allow for the cultural adaptation because there is a kind of openness that happens from a person who is not Hispanic leading it. There are suddenly a whole bunch of hidden expectations, if that person is Hispanic. Whereas that's going to get teased out if the person is not Hispanic. In some ways, the complete difference in culture from the person who was running it, but was still open, was actually more empowering (Medical interpreter #12; Exit interview).”
		Importance of cultural humility	“It's that openness that I think is even more important. Anybody's affect can be off putting if you think you're a know-it-all and I don't really care who you are now because now I don't really want to support you (laughter) and that'll shut me down. That was one of the reasons this kind of research is important because it's an interpreter who is doing the research. Yes, you happen to have another field, too, but this is for interpreters by interpreters and that's important (Medical interpreter #12; Exit interview).”
	Impact of the intervention	**Exemplar quote**	
		“… this information is freedom. This information is empowerment. And that's what we need to do… every single thing in our lives. We, most of the time, are doubtful or hesitant… and this is just saying, yes you are, and yes you can and this is the way and you are going to have the support and that is what this ancient teaching is giving us. I really liked it (Medical interpreter #9; Exit interview).”	
		“I did notice, not a change, but an impact. A positive impact of recognizing, of noticing, mostly noticing certain moments… I'm noticing myself, noticing how I'm feeling, noticing the feelings arising, noticing the emotions or the feelings in my body. For me that was huge, I'm like, ‘oh my God, I'm noticing. I'm recognizing, I'm thinking about it.' Before, I wasn't doing that. None of it (Medical interpreter #14; Exit interview).”	
	General feedback about content	**Sub-theme**	**Exemplar quote**
		Efforts to foster accountability in between sessions	I would have loved to see more activities for us to do on our own time, along with more engagement/accountability (like a log or journal), plus more debriefing time about how those activities went. This might necessitate additional meeting time, or perhaps an online discussion board. While I enjoyed the techniques discussed and our in-person meetings, it was too easy to forget to implement them between sessions (Medical interpreter #1; Free text survey item).

## 4. Discussion

By incorporating the insight of interpreting administrators and collaborators who have worked with medical interpreters, as well as the contributors to interpreter distress and psychosocial stressors from the relevant literature (Park et al., 2017; Lim et al., 2022), we adapted and piloted an MPIM program with medical interpreters using a single-arm pilot embedded QUAN(qual) mixed-methods study design (Schoonenboom and Burke Johnson, 2017). We hypothesized that effects would occur at the post-intervention time point and be maintained at 1-month follow-up; thus, significant differences were anticipated from pre- to post-intervention and from pre- to 1-month post-intervention. In support of our hypothesis, the overall scores for teamwork, coping, and resilience all increased from pre- to post-intervention and pre- to 1-month. As components of professional quality of life, compassion satisfaction and burnout were in partial support of our hypothesis demonstrating significant delayed effects of the intervention. Compassion satisfaction increased and burnout decreased from pre- to 1-month post-intervention; however, each had moderate effect sizes and improved when compared with available normative values (ProQOL, 2021) but were not statistically significant from pre- to post-intervention. Also, in partial support of our hypothesis, non-judging as a mindfulness component increased from pre- to post-intervention, however, was not sustained 1-month later. Stress demonstrated delayed moderate effects of the mindful practice from pre- to 1-month with a change from moderate to low perceived stress, and marginal statistical significance, however, was not significant from pre- to post-intervention. The intervention was unable to impact change in the mindfulness components of non-reactivity and acting with awareness and in the professional quality of life component of traumatic stress. Qualitative data revealed that those who attended every session had a strong *internal motivation* and that participation in the intervention helped to build involvement for those who may have been initially hesitant. Even though feelings were mixed about similar or larger *group size*, participants welcomed different perspectives from interpreters at different organizations and across different languages but wanted to limit the sessions to interpreters who practice within the same setting. As such, any future iterations should combine cohorts of medical interpreters. The impact of the intervention had positive effects, guiding participants to notice their thoughts, feelings, emotions, and physical sensations and be more responsive vs. reactive. Addressing fewer topics with more depth or the same number of topics but across multiple sessions or by extending the session duration was suggested; specifically, *more depth about professionalism* was requested. The role of the mindful practice facilitator with medical interpreters should be maintained by an interpreter with no specific requirement of race, ethnicity, or language, just *cultural humility* and openness. Finally, the integration of our quantitative and qualitative findings accentuated the positive impact of the mindful practice intervention.

Although there were differences in sampling demographics and methodology, our work adds to the previous work (Park et al., 2017) by demonstrating that an adapted mindful practice intervention was able to impact positive change in medical interpreter well-being. Group composition across the current and past study cohorts (Park et al., 2017) was similar with 53% and 54% identifying as Hispanic or Latino, respectively; however, Park et al. (2017) did not include medical sign language interpreters and had a greater diversity of medical spoken language interpreters. Park et al. (2017) delivered the CARE program for medical interpreters in one 4-h block, whereas the current study delivered a condensed MPIM program for 8 weeks with weekly in-person 1-h sessions. Stress, coping, and satisfaction were comparable outcome variables of interest across studies. Stress and coping were measured using the same tools; however, satisfaction was measured using different tools. We found a moderate effect of the intervention on stress was delayed and marginal statistical significance inviting confirmation with a larger study. This contrasted with Park et al. (2017) who found no differences in stress across time points (*p* = 0.360, *d* = 0.170). The mindful practice intervention was able to impact a significant increase in overall coping, whereas the relaxation response and resiliency program (Park et al., 2017) reported no overall change (p=0.130, *d*=0.330). We measured compassion satisfaction, which was defined as the pleasure derived from being able to work well (ProQOL, 2021) using the ProQOL, and Park et al. (2017) used modified items from the 2006 Massachusetts General Hospital staff survey to measure job satisfaction. Compassion satisfaction in the current study and job satisfaction in the past work (Park et al., 2017) both demonstrated significant improvement.

Future mindful practice intervention development and adaptation could borrow from some of the tactics used in the CARE program (Park et al., 2017). Participants in this study requested *presentation handouts*, in addition to electronic delivery of the presentation slides, to enhance their learning and reference later on as a reminder of what was learned. Participants also wished there were more *efforts to foster accountability in between sessions*. Even though each week concluded with an introduction of new practices or review of previous informal mindful practices that could be implemented between sessions, participants felt it was too easy to forget and were eager for more engagement, like a journal or a discussion board, and more group debriefing about their independent mindful practice efforts. As resources permit, a manual to write in during the sessions, inclusive of the didactic content and interactive exercises, as well as recordings to guide daily practice in between sessions for enhanced sustainment after completion of the intervention could be provided.

This study had a few limitations. Although we had interpreters from other medical organizations and combined medical sign and spoken language interpreter cohorts in our analyses, readers are cautioned about deriving generalizations using these limited data as the study may be underpowered. Additionally, because of the low response to the free text survey item and low participation in exit interviews, we recognize the limited degree to which the qualitative results represent the scope of reactions to the intervention. Because of the pilot nature of this study and our aim to detect trends, we elected not to control for type I errors across the multiple comparisons; thus, some false positives may exist. Investigators strongly encouraged medical interpreters to participate across the entire duration of the study; however, we did not exclude or withdraw those who missed any specific number of sessions. Data analyses did not compare across the number of sessions attended or compare across languages, years of medical interpreting, certification, or previous mindfulness experience because of our small sample. Past iterations of MPIM with physicians and medical educators have been conducted in Western New York, Norway, and the Netherlands with participants from Africa, Asia, Australia/Oceania, Europe, and North and South America (Epstein et al., 2022). With greater societal awareness, diversity, equity, and inclusion themes are increasingly incorporated into the MPIM content and cultural, racial, linguistic, and occupational diversity are increasingly being represented by the MPIM teaching faculty, facilitators, and learners. Future iterations of the mindful practice intervention with medical interpreters should further aspire to address linguistic and cultural diversity in the study population for broader representation and subsequent generalization. Formal contemplative practices of mindfulness are often inaccessible to deaf medical sign language interpreters or deaf patient communities because they encourage participants to soften or lower their gaze or close their eyes. This limits participation as deaf persons rely more on visual communication than their non-deaf (hearing) peers. To avoid the exclusion of minoritized groups, past studies have emphasized, that research participants and facilitators for mindfulness-based programs should be representative and have shown meaningful cultural adaptations in language, content, and methods (Castellanos et al., 2020; Eichel et al., 2021). The next steps in this research agenda will involve community collaborations to develop, disseminate, and assess accessible mindfulness resources (i.e., video recordings in sign language) as currently there are limited mindfulness opportunities available for deaf sign language users.

An array of training, resources, and support across intrapersonal, interpersonal, and organizational levels (Lim et al., 2022) are necessary for promoting the management of psychosocial stressors experienced by medical interpreters. Because communication access is a social determinant of health, protecting and promoting the health of medical interpreters are critical for ensuring equitable healthcare access for language-minority patients. Mindful practice could serve as an effective resource for medical interpreters when coping with work-related stressors. The most important findings from this study were improved and sustained teamwork, coping, and resilience, which replicated the improved and sustained well-being outcomes of the MPIM program with health professionals (Krasner et al., 2009; Epstein et al., 2022). All qualitative feedback gathered from study participants about intervention logistics and content will be incorporated into future iterations for improved intervention efficacy. After the completion of initial mindful practice training, any ongoing sessions should include members across disciplines, such as physicians and nurses to promote interpreters' *desired sense of belonging to the organization*. Efforts from this research demonstrate the need for greater visibility and attention to medical interpreters as essential members of the broader care team.

## Data availability statement

The raw data supporting the conclusions of this article will be made available by the authors, without undue reservation. Further inquiries can be directed to the corresponding author.

## Ethics statement

The studies involving humans were approved by University of Rochester, Research Subjects Review Board. The studies were conducted in accordance with the local legislation and institutional requirements. The Ethics Committee/Institutional Review Board waived the requirement of written informed consent for participation from the participants or the participants' legal guardians/next of kin because this study posed no greater than minimal risk. An information sheet was used instead when enrolling participants. Written informed consent was not obtained from the individual(s) for the publication of any potentially identifiable images or data included in this article because this study posed no greater than minimal risk.

## Author contributions

GR, RY-N, PV, AC, SB, and RE: conceptualization, visualization, and writing—review and editing. GR: data curation, project administration, and writing—original draft. GR and RY-N: formal analysis. GR, RY-N, AC, SB, and RE: methodology. All authors contributed to the article and approved the submitted version.

## References

[B1] Baer R. A. Smith G. T. Hopkins J. Krietemeyer J. Toney L. (2006). Using self-report assessment methods to explore facets of mindfulness. Assessment. 13, 27–45. 10.1177/1073191105283504 16443717

[B2] Beckman H. B. Wendland M. Mooney C. Krsner M. S. Quill T. E. Suchmann A. L.. (2012). The impact of a program in mindful communication on primary care physicians. Academic Medicine. 87:815–819. 10.1097/ACM.0b013e318253d3b2 22534599

[B3] Brach T. (2013). True Refuge: Finding Peace and Freedom in Your Own Awakened Heart. New York, NY: Bantam.

[B4] Brach T. (2022). Meditation, Emotional Healing, and Spiritual Awakening. Available online at: https://www.tarabrach.com/ (accessed on January 15, 2023).

[B5] Butow P. N. Lobb E. Jefford M. Goldstein D. Eisenbruch M. Girgis A.. (2012). A bridge between cultures: interpreters' perspectives of consultations with migrant oncology patients. Care Cancer. 20, 235–244. 10.1007/s00520-010-1046-z 21110046

[B6] Castellanos R. Yildiz Spinel M. Phan V. Aguayo R. O. Humphreys K. Flory K. (2020). A systematic review and mete-analysis of cultural adaptations of mindfulness-based interventions for Hispanic populations. Mindfulness 11, 317–322. 10.1007/s12671-019-01210-x

[B7] CCHI (2022). Candidate's Examination Handbook. Available online at: https://cchicertification.org/uploads/CCHI_Candidate_Examination_Handbook.pdf (accessed January 15, 2023).

[B8] CCIE (2019). Commission on Collegiate Interpreter Education Accreditation Standards. Available online at: http://www.ccie-accreditation.org/standards.html (accessed January 15, 2023).

[B9] Charon R. (2001). Narrative medicine. A model for empathy, reflection, profession, and trust. JAMA. 286, 1897–1902. 10.1001/jama.286.15.1897 11597295

[B10] Chozen Bays J. (2022). Mindful Medicine: 40 Simple Practices to Help Healthcare Professionals Heal Burnout and Reconnect to Purpose. Boulder, CO: Shambjala.

[B11] Cohen J. (1988). Statistical Power Analysis for the Behavioral Sciences. New York, NY: Routledge Academic.

[B12] Cohen S. Kamarck T. Mermelstein R. (1983). A global measure of perceived stress. J. Health Soc. Behav. 24:385–396. 10.2307/2136404 6668417

[B13] Cohen S. Williamson G. (1988). “Perceived stress in a probability sample of the United States,” in The Social Psychology of Health, ed. *Spacapan S, Oskamp S (SAGE)*, 31–68.

[B14] Columbia University Irving Medical Center (2022). Department of Medical Humanities and Ethics. Division of Narrative Medicine. Available online at: https://www.mhe.cuimc.columbia.edu/division-narrative-medicine (accessed January 1, 2023).

[B15] Cooperrider D. Whitney D. (2005). Appreciative Inquiry. A Positive Revolution in Change. San Francisco, CA: Berrett-Koehler Publishers.

[B16] Creamer E. G. (2018). An Introduction to Fully Integrated Mixed Methods Research. Thousand Oaks, C. A.: SAGE.

[B17] Ducar D. M. Penberthy J. K. Schorling J. B. Leavell V. A. Calland J. F. (2020). Mindfulness for healthcare providers fosters professional quality of life and mindful attention among emergency medical technicians. Explore 16, 61–68. 10.1016/j.explore.2019.07.015 31471216

[B18] Eichel K. Gawande R. Acabchuk R. L. Palitsky R. Chau S. Pham A.. (2021). A retrospective systematic review of diversity variables in mindfulness research, 2000–2016. Mindfulness 12, 2573–2592. 10.1007/s12671-021-01715-4

[B19] Epstein R. Krasner M. (2017). Mindful Practice^®^ Workshop Facilitator Manual. 3^rd^ Ed. Rochester, NY: University of Rochester Medical Center.

[B20] Epstein R. M. Marshall F. Sanders M. Krasner M. S. (2022). Effect of an intensive mindful practice workshop on patient-centered compassionate care, clinician well-being, work engagement, and teamwork. J. Contin. Educ. Health Prof. 42, 19–27. 10.1097/CEH.0000000000000379 34459443

[B21] Gale N. K. Heath G. Cameron E. Rashid S. Redwood S. (2013). Using the framework method for the analysis of qualitative data in multi-disciplinary health research. BMC Med. Res. Methodol. 13:117. 10.1186/1471-2288-13-117 24047204 PMC3848812

[B22] Geary C. Rosenthal S. L. (2011). Sustained impact of MBSR on stress, well-being, and daily spiritual experiences for 1 year in academic health care employees. J. Altern. Complement. Med. 17, 939–944. 10.1089/acm.2010.0335 22010779

[B23] Grabbe L. Higgins M. K. Baird M. Pfeiffer K. M. (2021). Impact of a resiliency training to support the mental well-being of front-line workers. Med. Care. 59, 616–621. 10.1097/MLR.0000000000001535 33827106 PMC8191373

[B24] Insight Dialogue Community (2022). Living Relational Dhamma. Available online at: https://insightdialogue.org/ (accessed January 15, 2023).

[B25] Irving J. A. Dobkin P. L. Park J. (2009). Cultivating mindfulness in health care professionals: a review of empirical studies of mindfulness-based stress reduction (MBSR). Complement. Ther. Clin. Pract. 15, 61–66. 10.1016/j.ctcp.2009.01.002 19341981

[B26] Kabat-Zinn J. (2013). Full Catastrophe Living (Revised Edition): Using the Wisdom of Your Body and Mind to Face Stress, Pain, and Illness. New York, NY: Bantam Books.

[B27] Kramer G. (2007). Insight Dialogue: The Interpersonal Path to Freedom. Boulder, CO: Shambhala Publications.

[B28] Krasner M. S. Epstein R. M. Beckman H. Suchman A. L. Chapman B. Mooney C. J.. (2009). Association of an educational program in mindful communication with burnout, empathy, and attitudes among primary care physicians. JAMA 302, 1284–1293. 10.1001/jama.2009.1384 19773563

[B29] Latif Z. Makuvire T. Feder S. L. Crocker J. Pinzon P. Q. Warraich H. J. (2022). Forgotten in the crowd: a qualitative study of medical interpreters' role in medical teams. J. Hosp. Med. 7, 719–725. 10.1002/jhm.12925 35912708

[B30] Lebares C. C. Hershberger A. O Guvva E. V. Desai A Mitchell J Shen W. (2018). Feasibility of formal mindfulness-based stress-resilience training among surgery interns. JAMA Surg. 153, e182734. 10.1001/jamasurg.2018.2734 30167655 PMC6233792

[B31] Leon A. C. Davis L. L. Kraemer H. C. (2011). The role and interpretation of pilot studies in clinical research. J. Psychiatr. Res. 45, 626–629. 10.1016/j.jpsychires.2010.10.008 21035130 PMC3081994

[B32] Lim P. S. Olen A. Carballido J. K. LiaBrateen B. Sinnen S. R. Balistrein K. A.. (2022). “We need a little help”: a qualitative study on distress and coping among pediatric medical interpreters. J. Hosp. Manage. Health Policy. 6, 36. 10.21037/jhmhp-22-23

[B33] Lomas T. Medina J. C. Ivtzan I. Rupprecht S. Eiroa-Orsa F. (2017). A systematic review of the impact of mindfulness on the well-being of healthcare professionals. J. Clin. Psychol. 74:319–355. 10.1002/jclp.22515 28752554

[B34] McCracken L. M. Yang S. (2008). A contextual cognitive-behavioral analysis of rehabilitation workers' health and well-being: influences of acceptance, mindfulness, and values-based action. Rehabil. Psychol. 53, 479–485. 10.1037/a0012854

[B35] McKee M. M. Barnett S. L. Block R. C. Pearson T. A. (2011). Impact of communication on preventive services among deaf American sign language users. Am. J. Prev. Med. 41, 75–79. 10.1016/j.amepre.2011.03.004 21665066 PMC3117257

[B36] Mehta D. H. Perez G. K. Traeger L. Park E. R. Goldman R. E. Haime V.. (2016). Building resiliency in a palliative care team: a pilot study. J. Pain Symptom Manage. 51, 604–608. 10.1016/j.jpainsymman.2015.10.013 26550936

[B37] MPIM (2022). Helping Clinicians Thrive. Available online at: https://mindfulpracticeinmedicine.com/ (accessed January 15, 2023).

[B38] Muzamil Kumar M. Ahmad Shah S. (2015). Psychometric properties of Podsakoff's organizational citizenship scale in the Asian context. Int. J. Indian Psychol. 3, 51–60. 10.25215/0301.152

[B39] NAD Website (2023). What is America Sign Language? Available online at: https://www.nad.org/resources/american-sign-language/what-is-american-sign-language/#:~:text=American%20Sign%20Language%20(ASL)%20is,important%20parts%20in%20conveying%20information (accessed on February 10, 2023).

[B40] NBCMI (2021). “Certified medical interpreter,” in NBCMI Candidate Handbook. Available online at: https://www.certifiedmedicalinterpreters.org/assets/docs/NBCMI_Handbook.pdf?v=20211117 (accessed January 15, 2023).

[B41] NCIEC (2023). Deaf Interpreter Institute. What is a Deaf interpreter? Available online at: http://diinstitute.org/what-is-the-deaf-interpreter/ (accessed on February 2, 2023).

[B42] Park E. R. Mutchler J. E. Perez G. Goldman R. E. Niles H. Haime V.. (2017). Coping and resiliency enhancement program (CARE): A pilot study for interpreters in cancer care. Psychooncology 26, 1181–1190. 10.1002/pon.4137 27196822 PMC5495620

[B43] Park E. R. Traeger L. Vranceanu A. M. Scult M. Lerner J. A. Benson H.. (2013). The development of a patient-centered program based on the relaxation response: the relaxation response resiliency program (3RP). Psychosomatics 54, 165–174. 10.1016/j.psym.2012.09.001 23352048

[B44] Pfau R. Steinbach M. Woll B. (2012). Sign Language An International Handbook. Berlin, Germany: De Gruyter Mouton.

[B45] ProQOL (2021). ProQOL Measure. Available online at: https://proqol.org/proqol-measure (accessed January 15, 2023).

[B46] RID (2023). Available Certifications. Available online at: https://rid.org/certification/available-certifications/ (accessed January 15, 2023).

[B47] Roat C. (1999). “Bridging the gap: a basic training for medical interpreters,” in Cross Cultural Health Care Program (CCHP) Seattle, WA. Available online at: https://xculture.org/bridging-the-gap/ (accessed February 10, 2023).

[B48] Roman G. Samar V. Ossip D. McKee M. Barnett S. Yousefi-Nooraie R. (2022a). The occupational health and safety of sign language interpreters working remotely during the COVID-19 pandemic. Prev. Chronic Dis. 19, 210462. 10.5888/pcd19.210462 35679479 PMC9258443

[B49] Roman G. Samar V. Ossip D. McKee M. Barnett S. Yousefi-Nooraie R. (2022b). Perspectives of sign language interpreters and interpreting administrators during the COVID-19 pandemic: A qualitative description. Public Health Rep. 138, 691–704. 10.1177/00333549231173941 37243519 PMC10225799

[B50] Sandelowski M. (2000). Whatever happened to qualitative description? Res. Nurs. Health. 23, 334–340. 10.1002/1098-240x(200008)23:4<334::aid-nur9>3.0.co;2-g 10940958

[B51] Sawilowsky S. S. (2009). New effect size rules of thumb. J. Moder. Appl. Stat. Methods. 8, 597–599. 10.22237/jmasm/1257035100

[B52] Schoonenboom J. Burke Johnson R. (2017). How to construct a mixed methods research design. Kolner Z. Soz. Sozpsychol. 69, 107–131. 10.1007/s11577-017-0454-1 28989188 PMC5602001

[B53] Smith B. W. Dalen J. Wiggins K. Tooley E. Christopher P. Bernard J. (2008). The brief resilience scale: assessing the ability to bounce back. Int. J. Behav. Med. 15(3):194–200. 10.1080/10705500802222972 18696313

[B54] Smith S. R. Chin N. P. (2012). “Social determinants of health in deaf communities,” in Public health – Social and Behavioral Health, ed. J. Maddock (Rijeka, Croatia: InTech), 449–460.

[B55] Spencer L. Richie J. Ormston R.. (2013). “Analysis: principles and processes,” in Qualitative Research Practice: A Guide for Social Science Students and Researchers, 2^*nd*^ ed, eds. J. Ritchie, J. Lewis, C. McNaughton Nicholls, and R. Ormston. (London: SAGE Publications, Ltd), 269–294.

[B56] The World Café (2008). Shaping Our Futures Through Conversations That Matter. The World Café Presents… A Quick Reference Guide for Putting Conversations to Work. Available online at: https://theworldcafe.com/ (accessed January 15, 2023).

[B57] Trowbridge K. Lawson L. M. (2016). Mindfulness-based interventions with social workers and the potential for enhanced patient-centered care: a systematic review of the literature. Soc. Work Health Care. 55, 101–124. 10.1080/00981389.2015.1094165 26745592

